# Poly (Vinyl Alcohol) Hydrogels Boosted with Cross-Linked Chitosan and Silver Nanoparticles for Efficient Adsorption of Congo Red and Crystal Violet Dyes

**DOI:** 10.3390/gels9110882

**Published:** 2023-11-07

**Authors:** Reem T. Alfuraydi, Nouf F. Al-Harby, Fahad M. Alminderej, Noura Y. Elmehbad, Nadia A. Mohamed

**Affiliations:** 1 Department of Chemistry, College of Science, Qassim University, Buraidah 51452, Saudi Arabia; hrbien@qu.edu.sa (N.F.A.-H.); f.alminderej@qu.edu.sa (F.M.A.); 2 Department of Chemistry, Faculty of Science and Arts, Najran University, Najran 55461, Saudi Arabia; nyalmehbad@nu.edu.sa; 3 Department of Chemistry, Faculty of Science, Cairo University, Giza 12613, Egypt

**Keywords:** PVA/chitosan blend-based hydrogels, cross-linking, silver nano bio-composites, adsorption studies, Congo Red dye, Crystal Violet dye

## Abstract

In our previous work, three different weight ratios of chitosan/PVA (1:3, 1:1, and 3:1) were blended and then cross-linked with trimellitic anhydride isothiocyanate (TAI) at a concentration depending on their chitosan content, obtaining three hydrogels symbolized by H_13_, H_11_, and H_31_. Pure chitosan was cross-linked with TAI, producing a hydrogel symbolized by H_10_. Further, three H_31_-based silver nanoparticles composites (H_31_/AgNPs1%, H_31_/AgNPs3%, and H_31_/AgNPs5%) were also synthesized. They were investigated, for the first time in this study, as adsorbents for Congo Red (CR) and Crystal Violet (CV) dyes. The removal efficiency of CR dye increased with increasing H_10_ content in the hydrogels, and with increasing AgNP content in the composites, reaching 99.91% for H_31_/AgNPs5%. For CV dye, the removal efficiency increased with the increase in the PVA content. Furthermore, the removal efficiency of CV dye increased with an increasing AgNP content, reaching 94.7% for H_31_/AgNPs5%. The adsorption capacity increased with the increase in both the initial dye concentration and temperature, while with an increasing pH it increased in the case of CV dye and decreased in the case of CR dye. The adsorption of CV dye demonstrated that the Freundlich isotherm model is better suited for the experimental results. Moreover, the results were best fitted with pseudo-second-order kinetic model.

## 1. Introduction

The drainage of the synthetic dye-containing effluents into the resources of water causes great anxiety because these dyes are very stable, highly toxic, and have the propensity for accumulation in the environment, threatening the life of both human beings and aquatic organisms. Contact with these dyes leads to conjunctivitis, asthma, rhinitis, and dermatitis, in addition to other allergy diseases [1]. Thus, these dyes should be removed before drainage into water resources, and this is considered one of the main universal environmental requirements [2].

Various methods have been applied for treating these effluents using ion exchangers, membranes for filtration, and bio-degradative, coagulating, flocculating, chemical precipitating, photo-degradative, oxidizing, and ozonizing agents [2,3]. Regardless of the good efficiency of these methods on the industrial level, there are some issues facing their application, such as the high consumption of energy, the high price of utilized chemicals, undesirable toxic by-products, and the poor performance of the dyes at a low concentration [3,4]. Nowadays, the adsorption method is a highly effective technique for dye removal due to its high efficiency, simplicity, rapidness, easy operation, low initial cost, adsorbent abundance, lack of undesirable secondary products, and the easy restoration of the adsorbents for reuse [5].

Poly(vinyl alcohol), PVA, an eco-friendly synthetic vinyl polymer, showed some unique physicochemical and mechanical features such as flexibility, water solubility, hydrophilicity, chemical stability, high tensile strength, inherent non-toxicity, non-carcinogenicity, bio-compatibility, bio-degradability, elasticity, and film and gel forming ability [6]. It has numerous biomedical, industrial and environmental applications since it is used as a basic material for replacing skin and the cartilage, vocal cord reconstruction, and contact lenses and as a better adsorbent of cationic dyes than anionic ones [7,8]. Thus, the enhancement of its adsorption capability toward anionic dyes is considered to be a desired approach for extending its application fields. Hence, its incorporation with another polymer with a high adsorption capacity for anionic dyes, such as chitosan, is deemed a key to the enhancement of its adsorption capability for anionic dyes.

Chitosan, an eco-friendly natural biopolymer, has various intriguing properties; it is renewable, abundant, non-toxic, highly viscous, a film forming-material, an antioxidant agent, an antimicrobial agent, a hypolipidemic agent, highly polyelectrolytic, a binder for metals, fats, and anionic dyes, bio-degradable, bio-compatible, and a wound healing accelerator. Thus, it could be utilized in the medicinal, industrial, and wastewater remediation fields [9,10,11]. Chitosan comes with a main issue, which is its high solubility in aqueous acids due to the formation of its protonated state. This restricts its use as an adsorbent for dyes since their effluents are usually acidic. Thus, it should be chemically modified to improve its stability in acidic media and make it a superb option as an adsorbent for dyes. Modification was achieved via the insertion of functional groups into its structure [12,13]; via grafting using functionalizing monomers [14]; through the polymer blending [15]; and via the incorporation of chemical cross-linkers [16,17,18].

Chitosan nanofiber membranes were fabricated by blending chitosan with PVA [15]. They were used as green adsorbents to withdraw CR dye from an aqueous solution. The adsorption capacity increased with an increase in the degree of deacetylation of chitosan. The Langmuir isotherm model and the pseudo-second-order kinetic model could accurately represent the adsorption process. This shows that chemical adsorption could be a rate-limiting phase and that 358 mg L^−1^ was the greatest adsorption capacity.

PVA/chitosan nanofiber mats improved with aluminum–cerium spinel oxide (CeAlO_3_) nanoparticles showed a better uptake for Methylene Blue (MB) dye, and was fitted to the pseudo-second-order kinetic model. The maximum adsorption capacity of MB dye was 817.81 and 714.61 mg/g for modified and virgin nanofibers, respectively [19]. The maximum adsorption capacity of a PVA/chitosan-based adsorbent film to remove Acid Orange 7 was 678 mg/g at 298 K and pH = 2.5. The adsorption was suitable to the Lagergren pseudo first-order kinetic model and was described using the Redlich–Peterson model [20].

A PVA/chitosan mixture nanofiber was used to adsorb Direct Red 80, Direct Red 81, and Reactive Red 180 dyes. The adsorption increased as the pH decreased and conformed to the pseudo-second-order kinetic model. This occurred at the specific homogeneous sites of the nanofiber blend (Langmuir model) [21].

Previously, we modified chitosan via a reaction of its -NH_2_ groups with TAI, achieving the inclusion of both the amide, thiourea, carboxylic groups in addition to the residual -NH_2_ groups in it. This remarkably boosted the removal of both the anionic CR and the cationic Basic Red 12 (BR 12) dyes by the obtained chitosan hydrogels [22,23,24]. Based on these findings, we have progressed in the preparation of another series of PVA/chitosan blend-based hydrogels. Both TAI and silver nanoparticles (AgNPs) were employed to develop their features as antimicrobial agents and as inhibitors for biofilm formation [25]. Thus, we anticipate that this series of PVA/chitosan blend-based hydrogels and AgNP bio-composites will possess a better adsorption capacity for both anionic and cationic dyes, according to their contents of PVA, cross-linked chitosan, and AgNPs. Therefore, in this research, our objective was to study the isotherm properties, kinetics, and optimization of the adsorption process of these hydrogels and AgNP bio-composites for the removal of CR and CV dyes. The effect of temperature, initial dye concentration, pH, PVA/cross-linked chitosan ratio (cross-linking moiety proportions), and AgNP content on the adsorption process was also evaluated. Further, the possibility of regenerating the prepared adsorbents for their reuse was investigated. 

## 2. Results and Discussion

### 2.1. Removal of the CR and CV Dyes

The expansion of the numerous industrial activities that mainly use dyes, such as those of the fabric, cosmetic, leather, food, printing, plastic, and paper industries, has resulted in the discharge of large amounts of colored effluents into the aquatic environment. This is a great public problem and one of the major threats not only to human beings but also to all aquatic creatures due to the hazardous nature of synthetic dyes, which is correlated with their high toxicity and high resistance against environmental factors such as heat, light, and microorganisms resulting from their complicated aromatic structures. Even low concentrations of these dyes lead to an unacceptable coloration of water, causing aesthetic problems, the prevention of light and oxygen from reaching aquatic living organisms, and the lowering of their photosynthetic activities, in addition to causing severe damage to the liver and the digestive and central nervous system in humans [26]. With respect to this concern, the removal of CR and CV dyes, as representative examples of anionic and the cationic dyes, respectively, from their aqueous solutions before they are discharged into the environment is one of the prime global environmental interests. Adsorbents based on chitosan showed a higher adsorption capacity for various dyes [27].

**Figure 1:**
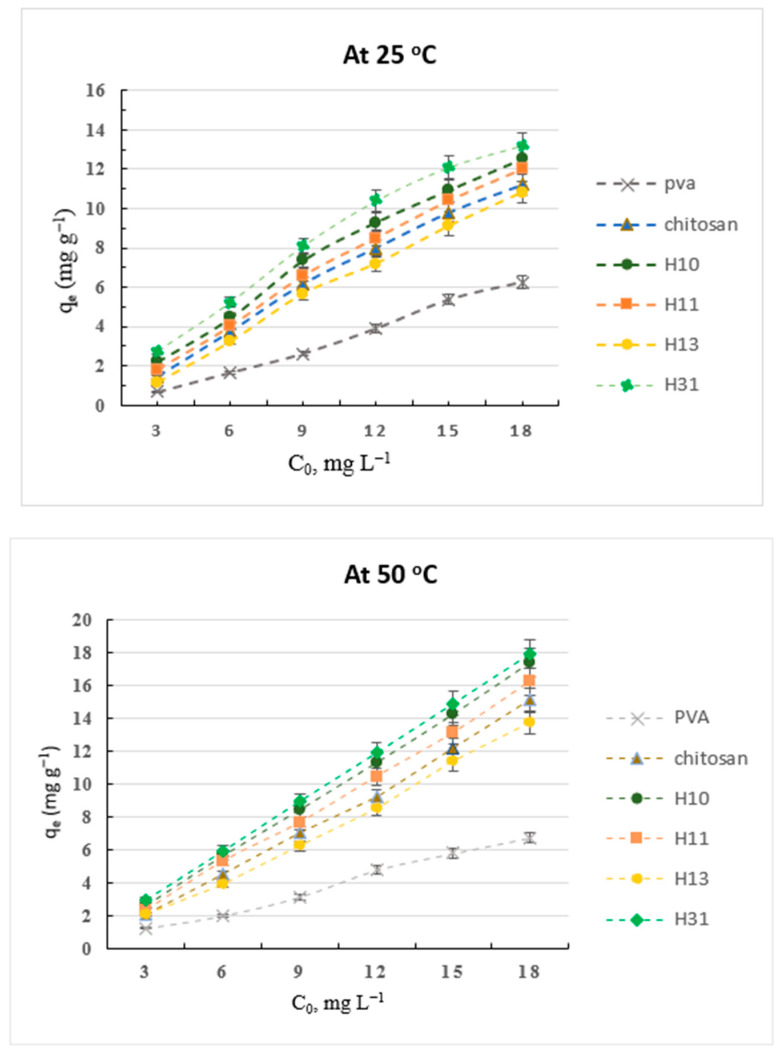
The capacity of CR dye to adsorb onto the prepared hydrogels at different dye concentrations, and at pH 7.

**Figure 2:**
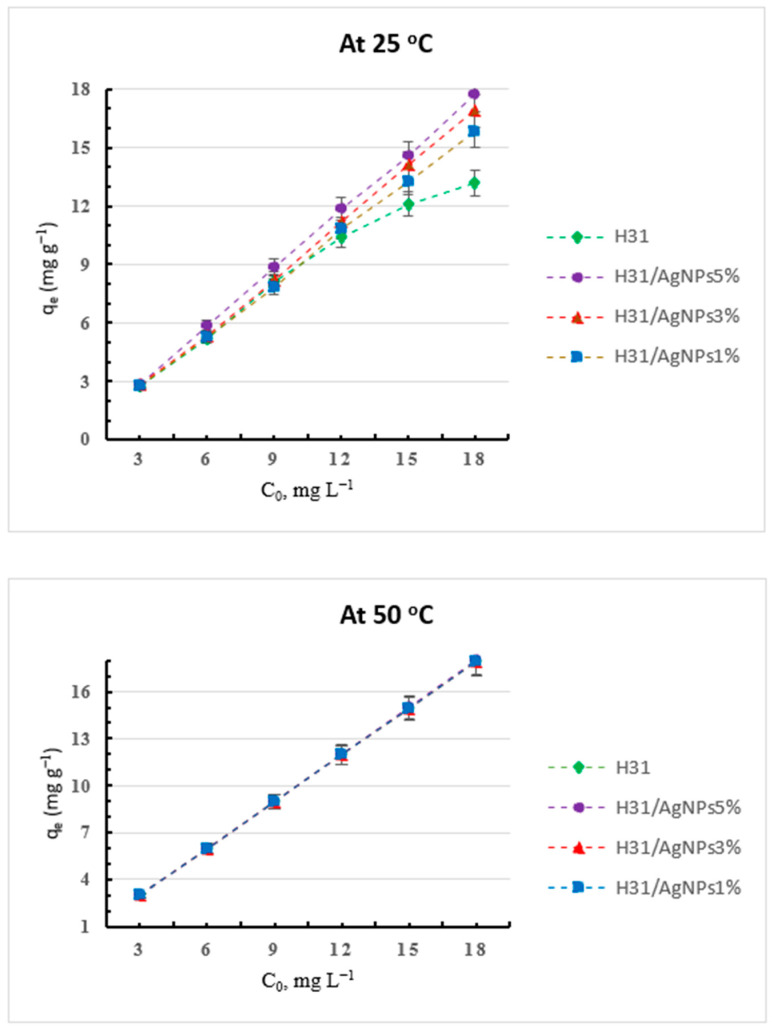
The capacity of CR dye to adsorb onto H_31_ and H_31_/AgNP composites at different dye concentrations, and at pH 7.

The results of the adsorption capacity (*q_e_*, Equation (1)) for different CR dye concentrations, at 25 and 50 °C, and at pH 7, for the prepared hydrogels and the H_31_/AgNP composites are shown in Figure 1 and Figure 2, respectively.

In accordance with the adsorption capacity, the effectiveness of the investigated hydrogels and the H_31_/AgNP composites for CR dye (Figure 1 and Figure 2) can be arranged as follows: PVA < H_13_ < chitosan < H_11_ < H_10_ < H_31_ < H_31_/AgNPs1% < H_31_/AgNPs3% < H_31_/AgNPs5%. For example, at a 18 mg L^−1^ dye concentration and at 25 °C and pH 7, the adsorption capacities for the CR dye of PVA, H_13_, chitosan, H_11_, H_10_, H_31_, H_31_/AgNPs1%, H_31_/AgNPs3%, and H_31_/AgNPs5% were 6.20, 10.80, 11.20, 12.00, 12.50, 13.20, 15.80, 16.90, and 17.74 mg g^−1^, respectively.

The % efficiency (Equation (3)) of the removal of CR dye by these hydrogels and the H_31_/AgNP composites at a 18 mg L^−1^ dye concentration, at 25 and 50 °C, and at pH 7 is summarized in Table 1. Notably, the % removal efficiency for CR dye can also be arranged as follows: PVA < H_13_ < chitosan < H_11_ < H_10_ < H_31_ < H_31_/AgNPs1% < H_31_/AgNPs3% < H_31_/AgNPs5%. For example, at a 18 mg L^−1^ dye concentration at 25 °C, and at pH 7 (Table 1), the % efficiency of the removal of CR dye by PVA, H_13_, chitosan, H_11_, H_10_, H_31_, H_31_/AgNPs1%, H_31_/AgNPs3%, and H_31_/AgNPs5% was 34.69, 60.00, 62.22, 66.70, 69.40, 73.33, 87.78, 93.89, and 98.54%, respectively.

PVA showed a lower adsorption capacity and a weaker removal efficiency for anionic CR dye than did chitosan due to the acidic and the basic nature of PVA and chitosan, respectively. On the other hand, H_10_ displayed a better adsorption capacity and a higher removal efficiency for CR dye than did chitosan due to the incorporation of additional basic groups (thiourea and amide linkages) into chitosan after its cross-linking process. Thus, the adsorption capacity and the removal efficiency of the prepared hydrogels for CR dye increased with an increasing H_10_ content, i.e., from H_13_ to H_31_. This is due to the increased number of incorporated thiourea and amide moieties into chitosan after the cross-linking process, from H_13_ to H_31_, which work as efficient binding sites for the CR anionic dye. Thus, the incorporation of H_10_ with PVA improves the adsorption capacity and the removal efficiency for CR dye, reaching their maximum in H_31_ [28]. Further, the dispersion of AgNPs into the matrices of H_31_ improved the adsorption capacity of the produced composites relative to that of H_31_ due to the increased surface area and the additional cross-linking between the electron-rich nitrogen and oxygen atoms of the hydrogel components and AgNPs [29,30].

**Figure 3:**
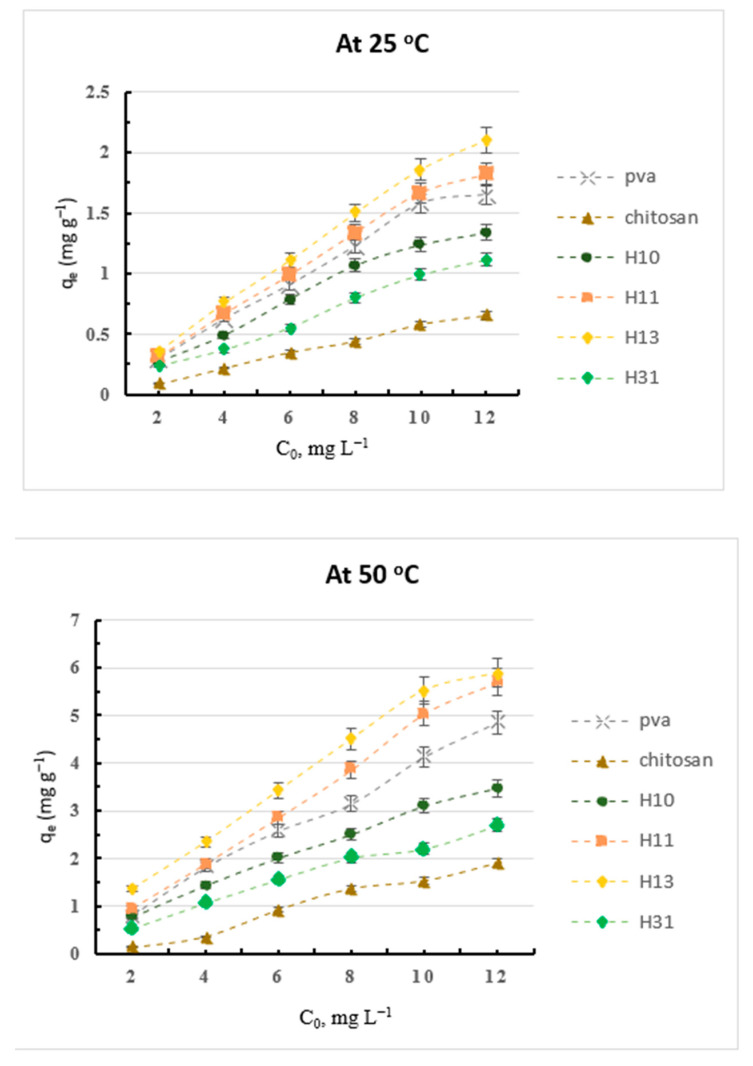
The adsorption capacity of CV dye onto the prepared hydrogels at different dye concentrations, and at pH 7.

**Figure 4:**
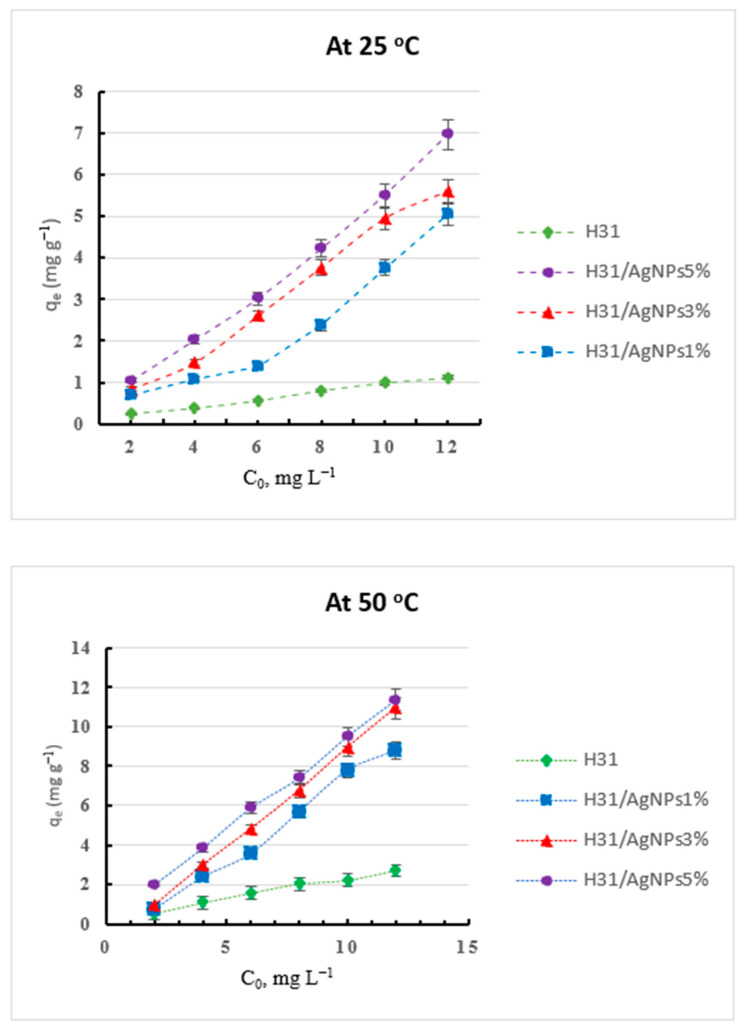
The adsorption capacity of the CV dye onto the H_31_ and H_31_/AgNP composites at different dye concentrations, and at pH 7.

The results of the capacity (*q_e_*, Equation (1)) for adsorption of the CV dye onto the prepared hydrogels and the H_31_/AgNP composites at different dye concentrations, at 25 and 50 °C, and at pH 7 are shown in Figure 3 and Figure 4, respectively.

Chitosan showed a lower adsorption capacity and a weaker removal efficiency for the basic CV dye than did PVA due to the basic and the acidic nature of chitosan and PVA, respectively, since PVA possesses a lot of hydroxyl groups which act as adsorption sites for the cationic dyes, while H_10_ displayed a better adsorption capacity and a higher removal efficiency for the CV dye than did chitosan due to the incorporation of the additional acidic carboxylic groups into chitosan, after its cross-linking process, which work as efficient binding sites for the CV cationic dye. Thus, the adsorption capacity and removal efficiency for the CV dye of the prepared hydrogels increased with the increase in their PVA content, i.e., from H_31_ to H_13_. Thus, the incorporation of PVA with H_10_ improved the adsorption capacity and the removal efficiency for CV dye, reaching the maximum in H_13_ [31]. Further, the loading of AgNPs inside the H_31_ matrices enhanced the adsorption efficiencies of the resulting composites in comparison to that of H_31_ due to the increased surface area, in addition to the many cross-linkages that originate between the AgNPs and the electron-rich nitrogen and oxygen atoms of the components of the hydrogels [29,30].

The adsorption capacity for both the CR and the CV dyes of the prepared hydrogels and the H_31_/AgNP composites are greatly dependent on the initial concentration of the dye. Their adsorption capacity increased with an increasing initial dye concentration from 3 to 18 mg L^−1^ and from 2 to 12 mg L^−1^ of the CR and the CV dyes, respectively (Figure 1, Figure 2, Figure 3 and Figure 4). For example, at 50 °C, the adsorption capacity of H_31_/AgNPs5% was 3.00, 5.96, 8.99, 11.96, 14.94, and 17.93 mg g^−1^ with the use of CR dye at initial concentrations of 3, 6, 9, 12, 15, and 18 mg L^−1^, respectively (Figure 2). Furthermore, at 50 °C, the adsorption capacity of H_31_/AgNPs5% was 1.97, 3.83, 5.88, 7.41, 9.51, and 11.37 mg g^−1^ when using CV dye at initial concentrations of 2, 4, 6, 8, 10, and 12 mg L^−1^, respectively (Figure 4). The impact of the initial concentration of the dye is based on the direct relationship between the dye’s concentration and the convenient sites on the surfaces of the adsorbents. An increase in the initial concentration of the dyes leads to an increase in the capacity of the adsorbents due to the great driving forces for the transfer of mass at a high initial concentration of the dye [32].

Contrary to the results of the CR anionic dye, in accordance with the adsorption capacity, at all the used temperatures and different dye concentrations, the effectiveness of the prepared hydrogels and the H_31_/AgNP composites toward the adsorption of the CV cationic dye (Figure 3 and Figure 4) can be classified as follows: chitosan < H_31_ < H_10_ < PVA < H_11_ < H_13_ < H_31_/AgNPs1% < H_31_/AgNPs3% < H_31_/AgNPs5%. For example, at a 12 mg L^−1^ dye concentration, at 50 °C, and at pH 7, the adsorption capacities for CV dye of chitosan, H_31_, H_10_, PVA, H_11,_ H_13,_ H_31_/AgNPs1%, H_31_/AgNPs3%, and H_31_/AgNPs5% were 1.91, 2.70, 3.47, 4.86, 5.70, 5.89, 8.78, 10.94, and 11.37 mg g^−1^, respectively. The % removal efficiency (Equation (3)) for CV dye of these hydrogels and the H_31_/AgNP composites at a 12 mg L^−1^ dye concentration, at 25 and 50 °C, and at pH 7 is summarized in Table 2. Notably, the % removal efficiency for CV dye of these hydrogels and the H_31_/AgNP composites can also be arranged as follows: chitosan < H_31_ < H_10_ < PVA < H_11_ < H_13_ < H_31_/AgNPs1% < H_31_/AgNPs3% < H_31_/AgNPs5%. For example, at a 12 mg L^−1^ dye concentration, at 50 °C, and at pH 7, the % removal efficiency for the CV dye of chitosan, H_31_, H_10_, PVA, H_11_, H_13_, H_31_/AgNPs1%, H_31_/AgNPs3%, and H_31_/AgNPs5% was 15.88, 22.50, 28.90, 40.46, 47.50, 49.08, 73.17, 91.18, and 94.71%, respectively.

Further, the capacity for the adsorption of CR and CV dyes onto the prepared hydrogels and the H_31_/AgNP composites increased with an increase in temperature from 25 to 50 °C (Figure 1 and Figure 4). This is ascribed to that an increase in the adsorption temperature, which enhanced the swelling ability inside the adsorbents, expediting dye molecule penetration inside the adsorbents. Further, the mobility of the dye molecules increased as the temperature of the adsorption process increased. This is well in agreement with the adsorption of CR dye by chitosan modified with terephthaloyl diisothiocyanate as a cross-linker [5]. 

**Figure 5:**
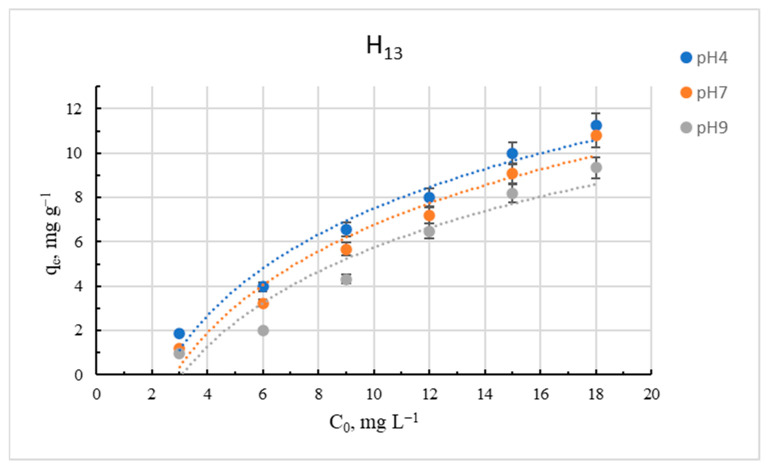
The capacity of CR dye to adsorb onto H_13_ at different dye concentrations, at different pHs, and at 25 °C.

**Scheme 1:**
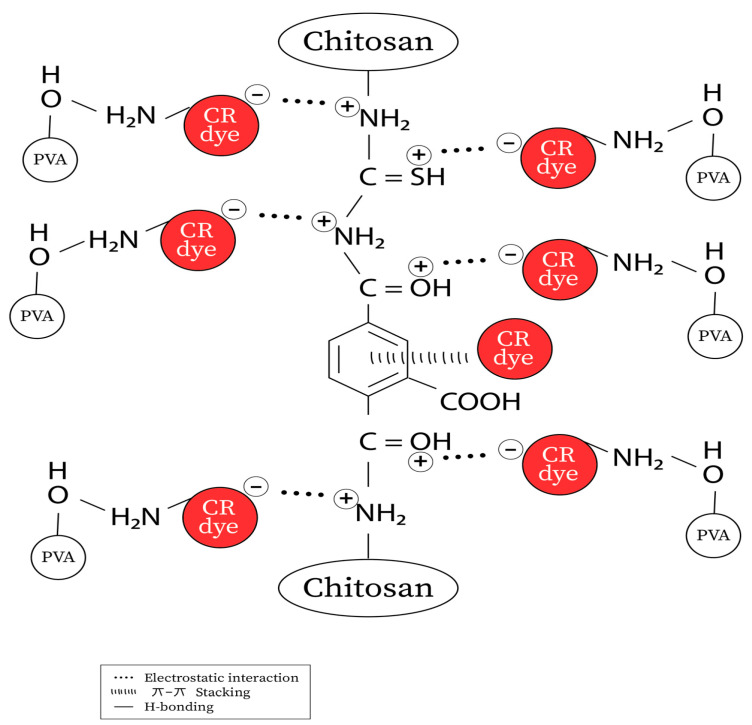
The proposed adsorption mechanism for CR dye of the prepared hydrogels at pH 4.

The impact of variance in the pH of the CR dye solution (4, 7, and 9) at 25 °C on the adsorption behavior (Equation (1)) of H_13_, as a representative example of the investigated hydrogels, is shown in Figure 5. The importance of the role that pH plays in the adsorption process was clearly revealed, because the adsorption capacity, *q_e_*, at pH 4 (the acidic medium) was higher than that at pH 7 and 9 (neutral and the basic media). This is ascribed to the fact that, in the acidic medium, the protonated groups of H_13_ (NH_3_^+^, NH_2_^+^, OH_2_^+^, C=OH^+^, and C=SH^+^) electrostatically interacted with the anionic groups of CR dye [33], in addition to π-π stacking resulting from the interaction between the π bonds of the aromatic rings of CR dye and the π bonds of the aromatic rings of cross-linking moieties, and the hydrogen bond formation between the amino groups of the CR dye and the hydroxyl groups of PVA, leading to a synergistic effect by which CR dye was adsorbed onto the prepared hydrogels as shown in Scheme 1. It is worth mentioning that the pHzpc value refers to the fact that the surface of the adsorbent had positive charges at low pH values (online Supplemental File S1).

**Figure 6:**
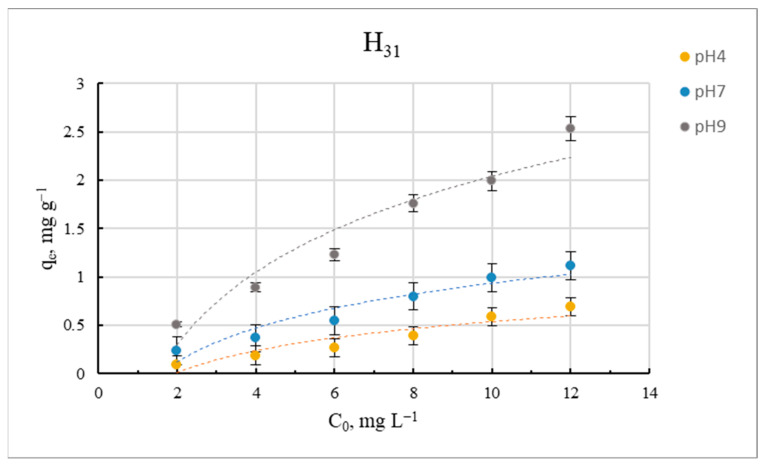
The capacity of CV dye to adsorb onto H_31_ at different dye concentrations, at different pHs, and at 25 °C.

**Scheme 2:**
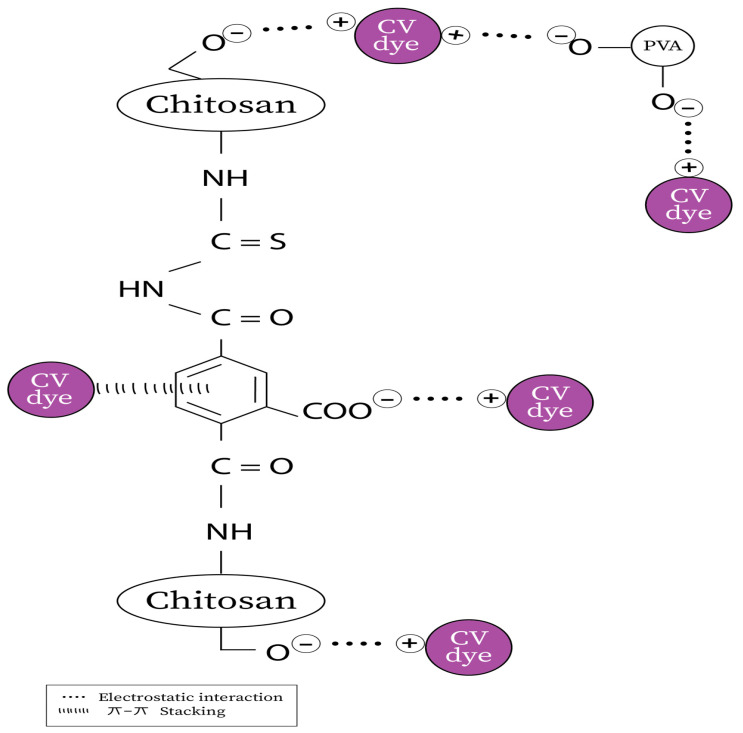
The proposed adsorption mechanism for CV dye of the prepared hydrogels at pH 9.

The effect of differences in the pH of the CV dye solution (4, 7, and 9) at 25 °C on the adsorption behavior (Equation (1)) of H_31_, as a representative example of the investigated hydrogels, is shown in Figure 6. It can be noted that the adsorption capacity, *q_e_*, at pH 9 (the basic medium) was higher than that at pH 7 and 4 (the neutral and the acidic media). This can be attributed to the fact that, in the basic medium, the deprotonated –COOH and −OH groups electrostatically interacted with the cationic groups of CV dye [34,35], in addition to π-π stacking resulting from the interaction between the π bonds of the aromatic rings of CV dye and the π bonds of the aromatic rings of the cross-linking moieties, leading to a synergistic effect by which CV dye was adsorbed onto the prepared hydrogels, as shown in Scheme 2. It is worth mentioning that the pHzpc value suggests that the surface of the adsorbent have negative charges at high pH values) online Supplemental File-S2).

### 2.2. Adsorption Isotherm Models

**Figure 7:**
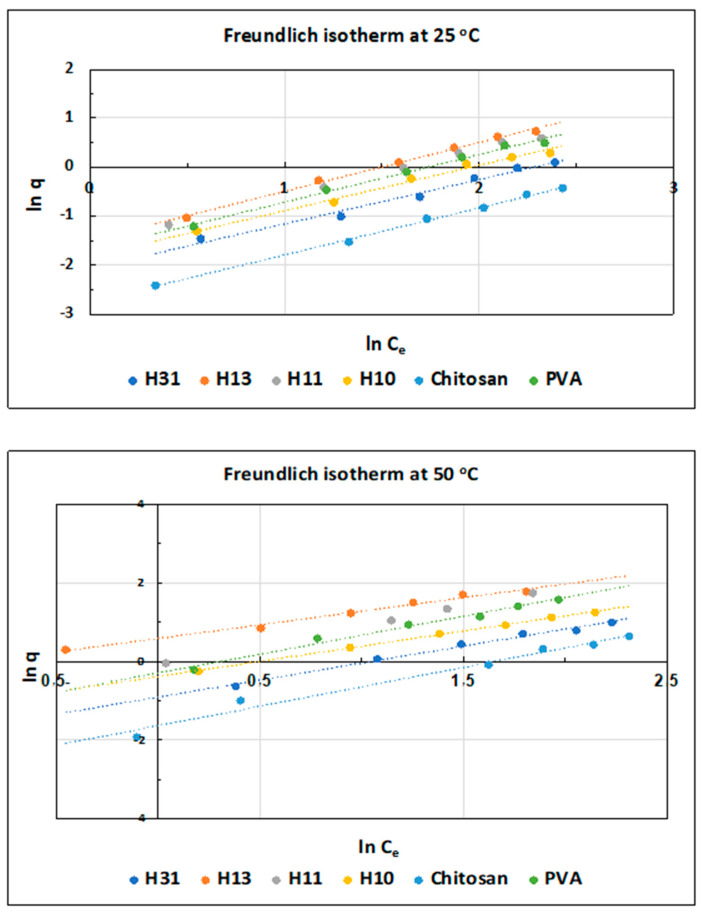
Freundlich isotherm of CV dye adsorption onto the prepared hydrogels.

**Figure 8:**
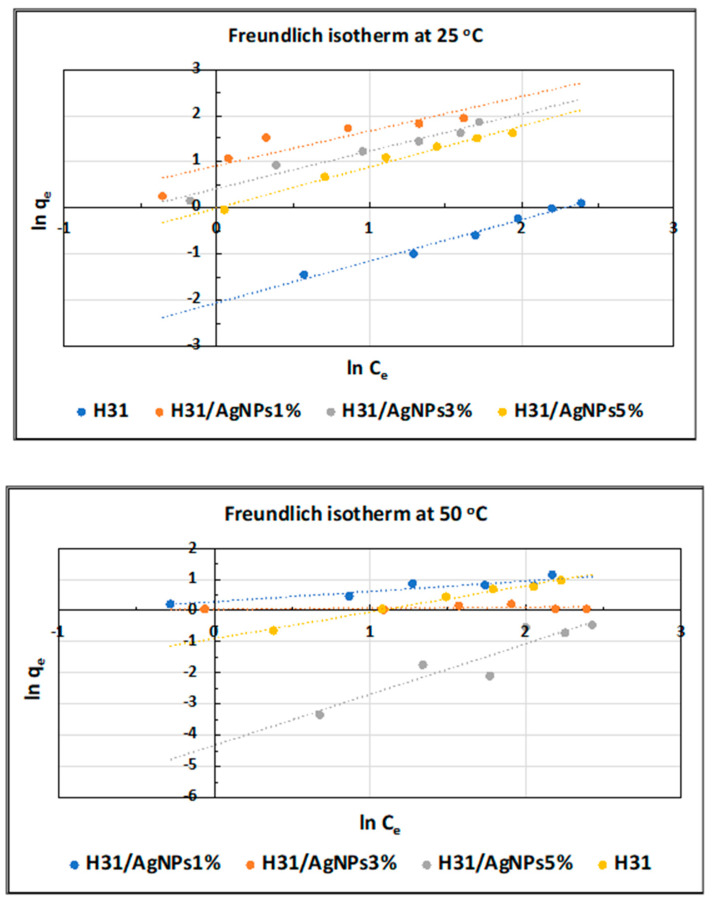
Freundlich isotherm of CV dye adsorption onto the H_31_ and the H_31_/AgNP composites.

Online Supplemental Files S3 and S4, and Figure 7 and Figure 8 show the Langmuir and the Freundlich adsorption isotherms of CV dye adsorbed onto the prepared hydrogels and the H_31_/AgNP composites, and the adsorption isotherm parameters are listed in Table 3 and Table 4. The regression coefficient (R^2^) values of the hydrogels and the H_31_/AgNP composites obtained from the Freundlich model at 50 °C ranged from 0.893 to 0.999 and from 0.857 to 0.863, respectively, which were much higher than those obtained from the Langmuir model, which ranged from 0.010 to 0.891 for the hydrogels and from 0.067 to 0.757 for the H_31_/AgNP composites. This indicates that the Freundlich model fitted better to the experimental results since the Freundlich isotherm assumes that multilayer adsorption and the adsorption occurs on heterogeneous surfaces [36]. The 1/*n* values for the hydrogels (0.69–0.99) indicated that this isotherm was favorabl while the values of 1/*n* for the H_31_/AgNP composites were greater than unity, evidencing a favorable adsorption of the CV dye by the H_31_/AgNP composites, which could have occurred due to cooperative adsorption among the active sites with the different adsorption capacities (energies). Thus, there are considerable interactions among the solute molecules, increasing the adsorption efficiency with the increase in the solute concentration in the solution. This reinforces the belief that adsorption possibly occurs on multiple layers, which is obvious proof of mostly a physical adsorption mechanism [37].

### 2.3. Adsorption Kinetics

**Figure 9:**
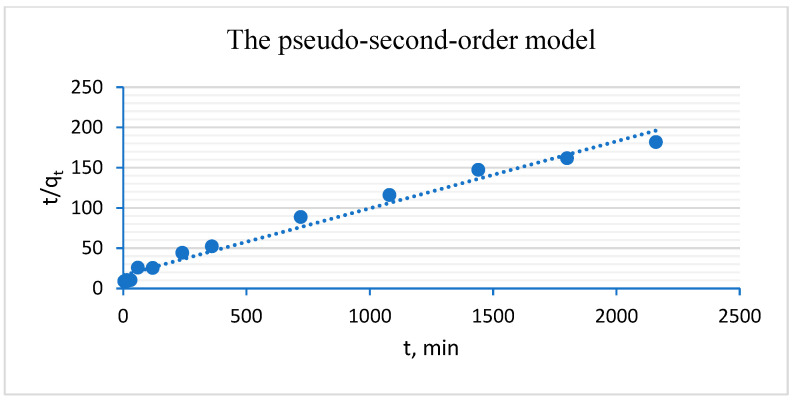
A plot of linear regressions of the pseudo-second-order kinetic model for the adsorption of CR dye onto H_31_.

**Figure 10:**
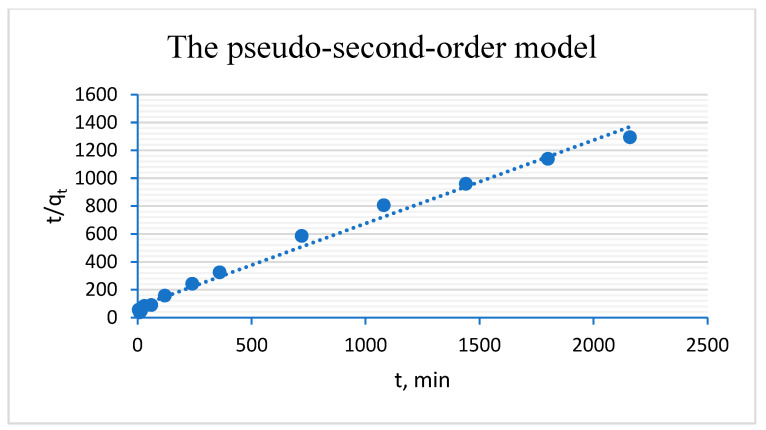
A plot of the linear regressions of the pseudo-second-order kinetic model for the adsorption of CV dye onto H_13_.

The kinetic outcomes of the adsorption of CR dye by H_31_ and that of CV dye by H_13_ are listed in Table 5. The adsorption plots of the pseudo-first order and the pseudo-second order are illustrated in online Supplemental Files S5 and S6 and Figure 9 and Figure 10, respectively.

The pseudo-second-order model showed a higher value of correlation coefficients, R^2^, (0.980 for H_31_ and 0.990 for H_13_) than did the pseudo-first order model (0.970 for H_31_ and 0.959 for H_13_). This is owed to the fact that the pseudo-second-order model shows a typical fit for describing the adsorption of both CR and the CV dyes onto the H_31_ and the H_13_ surfaces. In addition, there is good consistence between the values of the experimental and calculated *q_e_* for the pseudo-second-order model, which are equal to 13.45 and 12.00 mg g^−1^, respectively, for the adsorption of CR onto H_31_, and are equal to 1.67 and 1.67 mg g^−1^, respectively, for the adsorption of CV dye onto H_13_. This indicates and emphasizes the outstanding fit of the pseudo-second-order kinetic model for the adsorption of CR and CV dyes by H_31_ and H_13_, respectively.

### 2.4. Desorption Studies

The ability to reuse adsorbents is deemed to be one of the most significant characteristics that reduces the costs of adsorption techniques via the renewal of adsorbents [12].

**Figure 11:**
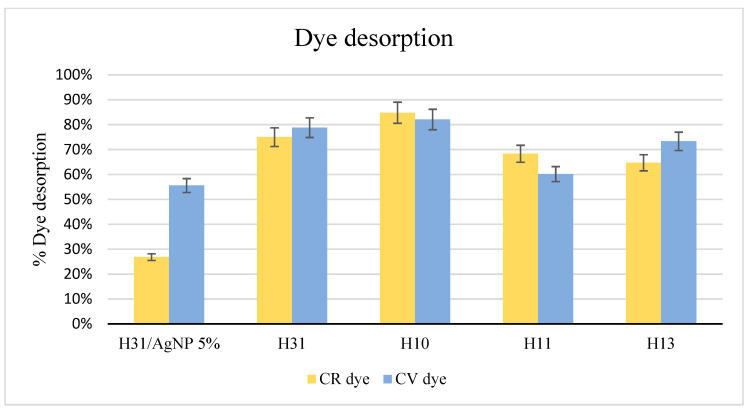
Desorption percentages of CR and the CV dyes from the adsorbents.

The desorption of the CR and the CV dyes was calculated using Equation (14). Methanol, as a desorption medium, showed that the desorption percentage reached 75 and 79% for the adsorption of CR and CV dyes onto H_31_, respectively, and reached 65 and 73% for the adsorption of CR and CV onto H_13_, respectively (Figure 11). These outcomes proved that the hydrogels could be effectively reused to adsorb both the CR and the CV dyes from their aqueous solutions.

### 2.5. Comparison of Sorption Capacity for CR and CV Dyes onto Various Adsorbents

To identify the performance of the synthesized hydrogels and the H_31_/AgNP composites, in the present study, their maximum adsorption capacities for both the CR and CV dyes were compared with those of other adsorbents, as shown in Table 6 and Table 7, respectively. The investigated hydrogels and the H_31_/AgNPs5% composite showed an adsorption capacity for CR dye that was better than that of some of the reported adsorbents [33,38,39,40,41,42], and that was lower than that of others [43,44,45,46] listed in Table 6. This reflects the prospects of boosting the adsorption capacity of PVA for CR dye via blending it with cross-linked chitosan and dispersing AgNPs into its matrices. This is due to the incorporation of a variety of cationized functional moieties during the cross-linking process which act as sites for binding the anionic CR dye. Thus, it is concluded that the synthesized hydrogels and the H_31_/AgNP composites can be considered favorable adsorbents for CR dye removal. On the other hand, in the case of CV dye, the adsorption capacity of the synthesized hydrogels and the H_31_/AgNPs5% composite was less than that of the previously reported adsorbents (Table 7) [47,48,49,50,51,52,53].

## 3. Conclusions

The adsorption capacity and the % removal efficiency for CR dye of the investigated hydrogels and the H_31_/AgNP composites increased with the increase in their H_10_ and AgNP content, and can be arranged as follows: H_31_/AgNPs5% > H_31_/AgNPs3% > H_31_/AgNPs1% > H_31_ > H_10_ > H_11_ > chitosan > H_13_ > PVA. This is due to the increased number of incorporated thiourea and amide moieties in chitosan after the cross-linking process, from H_13_ to H_31_, which work as efficient binding sites for CR anionic dye via electrostatic interaction and hydrogen bond formation. Contrary to the results for the CR anionic dye, the capacity for the CV cationic dye to adsorb onto the prepared hydrogels and the H_31_/AgNP composites can be classified as follows: H_31_/AgNPs5% > H_31_/AgNPs3% > H_31_/AgNPs1% > H_13_ > H_11_ > PVA> H_10_ > H_31_ > chitosan. Thus, the adsorption capacity and the removal efficiency for CV dye of the prepared hydrogels increased with an increase in their PVA content, i.e., from H_31_ to H_13_. Further, the loading of silver nanoparticles inside the H_31_ matrices enhanced the adsorption efficiencies of the resulting composites in comparison to those of the H_31_ due to the increased surface area, in addition to the many cross-linkages that originated between the AgNPs and the electron-rich nitrogen and oxygen atoms of the components of the hydrogels. The adsorption capacity for both the CR and the CV dyes of the prepared hydrogels and the H_31_/AgNP composites is greatly dependent on the initial dye concentration, the temperature, and the initial pH of the dye solution. The adsorption kinetics were effectively explained using a pseudo-second-order model for both the dyes. The Freundlich isotherm model fitted better to the experimental results of the adsorption of the CV dye onto the prepared hydrogels and the H_31_/AgNP composites, indicating that multilayer adsorption occurred on the heterogeneous surfaces. Thus, one can conclude that the incorporation of PVA, H_10_, and AgNPs in the same hydrogel matrix considerably enhances the PVA adsorption capacity for CR and the CV dyes, depending on the content of both H_10_ and the AgNPs in the hydrogel. Thus, it is possible to synthesize PVA hydrogels with good adsorption capabilities for cationic and anionic dyes via the adjustment of the H_10_ and AgNP content in these hydrogels. This is a suitable approach with which to attain promising materials that can efficiently compete with traditional adsorbents. 

## 4. Materials and Methods

### 4.1. Materials

Chitosan, with a deacetylation degree of 88.2% and a molecular mass of 2.9–3.1 × 10^5^ g mol^−1^, was supplied by Funakoshi Co. Ltd., (Tokyo, Japan). Ammonium thiocyanate (99.99%), silver nitrate (>99%), polyethylene glycol-400, trisodium citrate (≥99%), and poly (vinyl alcohol), PVA, with a degree of hydrolysis of 99.0–99.8% and molecular mass of 8.9–9.8 × 10^4^ g mol^−1^, were purchased from Sigma-Aldrich (Saint Louis, MO, USA). Trimellitic anhydride chloride (98%) was provided by Aldrich (Darmstadt, Germany). Congo Red (CR, ≥98, λ_max_ = 497 nm) and Crystal Violet (CV, ≥98, λ_max_ = 600 nm) dyes were obtained from Winlab (Leicestershire, UK). Online Supplemental File S7 shows the chemical structure of both the dyes, and their calibration curves.

### 4.2. Preparation of Chitosan/PVA Hydrogels

**Scheme 3:**
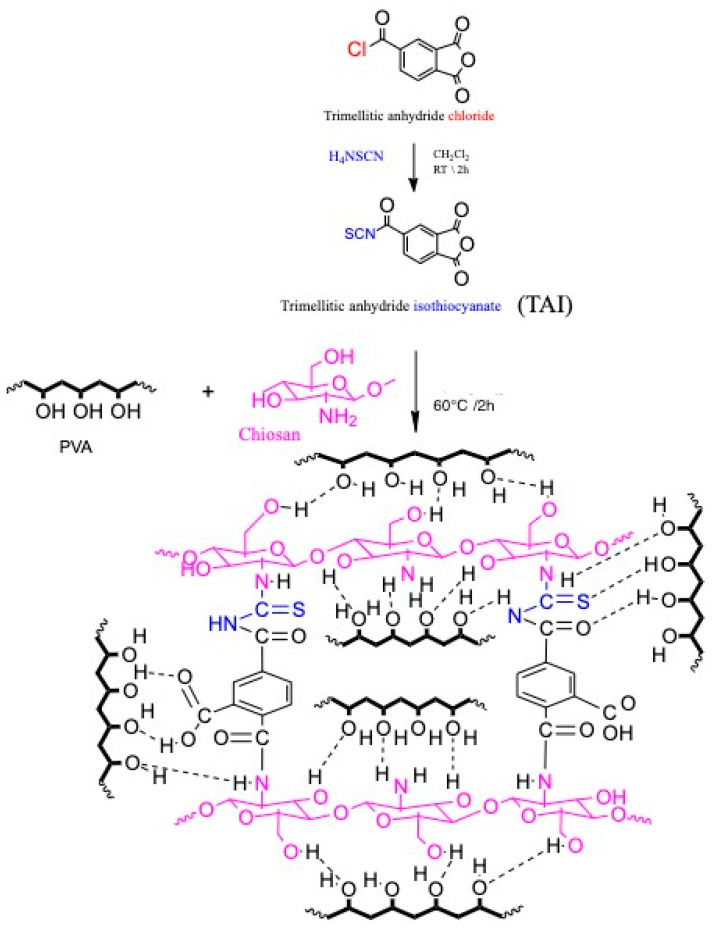
PVA/chitosan blend-based hydrogels.

Following the procedure reported in our previous work [25], firstly, a prespecified molar quantity of solid trimellitic anhydride chloride was gradually added to an equimolar quantity of NH_4_SCN that was dissolved in 25 mL of CH_2_Cl_2_, followed by the addition of polyethylene glycol-400 (1 mL), a phase transfer agent, stirring this well at 25 °C for 120 min. The reaction mixture was filtered to isolate the white precipitate (the by-product, NH_4_Cl) and obtain the trimellitic anhydride isothiocyanate cross-linker (TAI, Scheme 3) as a filtrate [25]. Secondly, a chitosan solution (1.5% wt/v using aqueous acetic acid (1% *v*/*v*) as a solvent) was blended with a PVA solution (5.0% wt/v, in distilled water at 60 °C), stirring this well at 60 °C for 60 min. TAI was slowly added to the resulting chitosan/PVA blend solutions with various weight ratios (Table 8), stirring this well at 60 °C for 120 min, and then at room temperature overnight. The molar ratio of TAI to the chitosan component in the blend was 1:2, respectively (Table 8 and Scheme 3). The acetic acid was eliminated from the resulting hydrogels via a neutralizing reaction using a NaHCO_3_ solution, then soaked in methanol for dewatering and desalting, filtration, and finally drying at 60 °C. The obtained hydrogels showed a color ranging from faint yellow to dark yellow in accordance with the TAI content that was based on the content of chitosan in the hydrogel. The ratios of the reactants are summarized in Table 8, with the production of four hydrogels symbolized as H_10_, H_11_, H_13_, and H_31_ [25].

### 4.3. Preparation of H_31_/Silver Nanoparticle (H_31_/AgNP) Composites

**Scheme 4:**
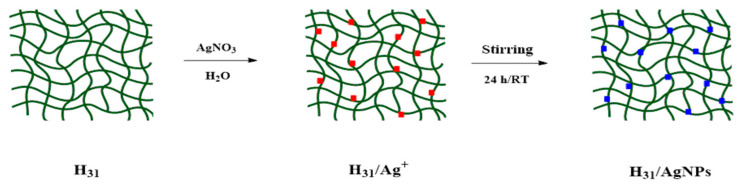
Synthesis of H_31_/AgNP composite.

Three different predetermined quantities of AgNO_3_ were separately dissolved in 10 mL of deionized H_2_O. Each AgNO_3_ solution was stirred with a constant weight of H_31_ (1 g) that priorly swelled in 50 mL of trisodium citrate solution (0.01 mol L^−1^) at room temperature for 24 h. A perceivable color variation in the three producing composites was detected that ranged from light to dark brown in proportion to the increase in the AgNO_3_ concentration. This indicated that the Ag^+^ cations were reduced to AgNPs inside the H_31_ matrix (Scheme 4). The composites were filtered, rinsed repeatedly with water then methanol, and dried at 60 °C. The used concentrations of silver nitrate were 1 wt%, 3 wt%, and 5 wt% in accordance with the weight of H_31_, producing three composites symbolized as H_31_/AgNPs1%, H_31_/AgNPs3%, and H_31_/AgNPs5%, respectively [25].

### 4.4. Characterization of Chitosan/PVA Hydrogels and H_31_/AgNP Composites

The structure and morphology of the prepared hydrogels and H_31_/AgNP composites were confirmed using various analytical techniques: elemental (Perkin Elmer C, H, N, S Analyzer, Model 2410 series II, Waltham, MA, USA), FTIR (Agilent Technologies FTIR Spectrometer, Cary 600 Series, Santa Clara, CA, USA), XPS (Kratos XSAM Spectrometer, Model 800, Manchester, UK), XRD (advanced wide-angle X -ray diffractometer (Brucker’s D-8), SEM (scanning electron microscope, Jeol-JSM-6060LV), EDS (scanning electron microscope (Quanta FEG 250) equipped with an energy-dispersive X-ray spectrometer) and TEM (transmission electron microscope (JEM-HR-JEOL-JEM2100) analyses. The results, shown in online Supplemental File S8, were well in agreement with those reported in our previous work [25].

Table 8 illustrates that the percentage of nitrogen and sulfur increased with increasing H_10_ content in the hydrogels (from H_13_ to H_31_), while the percent of carbon, hydrogen, and oxygen increased with the increasing PVA content in the hydrogels (from H_31_ to H_13_), indicating the successful preparation of chitosan/PVA hydrogels.

In comparison to the FTIR spectra of chitosan and PVA, shown in online Supplemental File-S8 (1), all the prepared hydrogels showed similar spectra that were characterized by the disappearance of the doublet peak of the primary amine groups of chitosan, and the appearance of a new peak around 1717 cm^−1^ attributed to **CO**OH in the cross-linking linkages. The peak of CO**OH** in the cross-linking linkages overlapped with that of the NH groups. The overlap between the peaks of the −**CO**NH, NH (secondary amide), and aromatic C=C bonds resulted in the appearance of a new peak near 1656 cm^−1^. The peak of the C=S groups that overlapped with the stretching vibration of C-O appeared as a strong broad peak around 1085 cm^−1^. The bending vibration peaks of N-C-S groups were observed near 1424 and 588 cm^−1^. The intensity of these peaks increased with an increasing cross-linker linkage content and consequently with the increasing chitosan content in the prepared hydrogels, i.e., from H_13_ to H_31_. Moreover, the FTIR spectra of H_31_/AgNPs3% and H_31_/AgNPs5%, shown in online Supplemental File S8 (2), displayed additional peaks in the range of 500–800 cm^−1^, indicating AgNP formation inside the composites.

The XPS spectrum of the H_31_/AgNPs5% composite, shown in online Supplemental File S8 (3), proves the formation of AgNPs. It shows two peaks at 368.2 and 374.0 eV which are consistent with 3d_5/2_ and 3d_3/2_ for silver metal. The other two peaks at 370.3 and 376.1 eV could be attributed to the complex resulting from the interaction of silver with the carbonyl and hydroxyl groups in H_31_.

Compared to the XRD pattern of virgin chitosan, shown in online Supplemental File S8 (4), which exhibited two peaks around 2θ = 10° and 20°, corresponding to its amorphous and crystalline fractions, respectively, that of H_10_ showed an increase in its amorphous fraction and a decrease in its crystalline fraction. This is due to the consumption of the hydrogen bond-forming functional groups during the cross-linking process. Virgin PVA showed three peaks: around 2θ = 10° (very weak and broad), near 2θ = 20° (very intensive and sharp), and around 2θ = 40° (weak and broad). H_13_, H_11_, and H_31_ displayed three relatively amorphous peaks around 2θ = 10°, 20°, and 40°. Their intensities and sharpness increased with an increase in the PVA proportion in the hydrogels (from H_31_ to H_13_).

The generation of AgNPs in the matrices of H_31_, as shown in online Supplemental File S8 (5), was confirmed via XRD analysis, which showed some characteristic crystalline peaks near 2θ 38.18°, 44.25°, 64.55°, 77.58°, and 81.81° with plane distances of 2.357°A, 2.039°A, 1.444°A, 1.229°A, and 1.165°A, respectively, which are consistent with those of the pure Ag metal. Their intensity increased with an increase in the AgNP content in H_31_. These peaks were indexed to the crystal planes of (111), (200), (220), (311), and (222) of face-centered cubic (fcc) Ag.

SEM images of chitosan and PVA, shown in online Supplemental File S8 (6), showed smooth surfaces, while all the prepared hydrogels had rough surfaces full of grooves and containing a lot of homogeneously distributed pores resulting from the cross-linking process, resulting in very open structures with increased surface areas. These pores facilitated the penetration of water into the hydrogel and acted as sites of interaction between the external stimuli and the hydrophilic polar groups of the hydrogels. The pores were denser and more regular with an increasing H_10_ content in the hydrogel due to the increased concentration of the used cross-linker.

The in situ-created AgNPs were homogeneously distributed without agglomeration or aggregation as bright spots into H_31_/AgNP composites, as shown via the SEM images in online Supplemental File S8 (7). This is owed to the existence of a lot of marked capping groups onH_31_, acting as efficient ligands for the stabilization of the formed AgNPs. Moreover, the EDS diagram of H_31_/AgNPs3% showed a peak corresponding to that of silver of 5.98 wt%, indicating the considerable adsorption of AgNPs onto H_31_. 

TEM images of H_31_/AgNPs5% composites at different magnifications, shown in online Supplemental File S8 (8), illustrated the uniform dispersion of the AgNPs as spherically shaped spots of sizes ranging between 13 and 26 nm.

### 4.5. pH of Zero-Point Charge—pHzpc

The pHzpc is a significant parameter that enables thorough comprehension of surface adsorption mechanisms. To determine the pHzpc, 10 mg of the hydrogel was stirred overnight in 10 mL of the NaCl solutions (0.01 M) at initial assorted pH values varying between 2 and 10. The pH values of these solutions were adjusted utilizing HCl/NaOH solutions (0.1 M). The pHzpc is determined as the pH value at ΔpH = 0 [24].

### 4.6. Batch Adsorption Tests

Congo Red (CR) and Crystal Violet (CV) dyes were selected in this study as representative examples for the anionic and the cationic dyes, respectively, due to their large-scale use in various dyeing processes.

A series of batch tests proceeded for determining the adsorption capability of both CR and CV dyes onto the prepared hydrogels and H_31_/AgNP composite adsorbents. The experiment on dye adsorption was carried out using 10 mg of the adsorbent in 10 mL of an aqueous dye solution of concentrations ranging from 2 to 18 mg L^−1^, at 25 and 50 °C, at pH 4, 7, and 9, and under a shaking speed of 80 rpm for 48 h, at which point an equilibrium in all the measurements was attained. HCl and NaOH were used to adjust the pH of the dye solutions. The unabsorbed portion of the dyes in the solution was spectrometrically determined using a Shimadzu UV/Vis 1650 spectrophotometer at 497 nm for CR dye and at 600 nm for CV dye. 

The adsorption capacity and the % removal efficiency of the adsorbent for the dyes were calculated based on Equations (1)–(3) [12]:

(1) $$q_{e}=\frac{\left(C_{o-}C_{e}\right)xV}{W}$$

(2) $$q_{t}=\frac{\left(C_{o-}C_{t}\right)xV}{W}$$

(3) $$\%\;\mathrm{Removal}\;\mathrm{efficiency}=(\frac{\left(C_{o-}C_{e}\right)}{C_{o}})\times 100$$

where *q_e_* and *q_t_* (mg g^−1^) are the amount of the adsorbed dye at the equilibrium time and t time, respectively, *C_o_* (mg L^−1^) is the initial concentration of the dye, *C_e_* (mg L^−1^) is the concentration of the dye at the equilibrium time, *V* (L) is the volume of the dye solution, and *W* (g) is the weight of the adsorbent. 

### 4.7. Adsorption Isotherms

The adsorption isotherm indicates the relation that associates the quantity of the solute adsorbed by the adsorbent and the solute concentration in the solution at the equilibrium at a certain temperature.

#### Types of Adsorption Isotherm Equations

In the last decade, several equilibrium isotherm models have been established, such as the Brunauer-Emmett-Teller, Dubinin-Radushkevich, Koble-Corrigan, Flory–Huggins, Redlich–Peterson, Radke–Prausnitz, Khan, Temkin, Toth, Hill, Sips, Langmuir, and Freundlich isotherm models. These models were derived in accordance with three essential advances: (i) kinetic considerations where the rates of adsorption and desorption are similar, (ii) the thermodynamic basis, and (iii) the potential theory that commonly communicates a major idea in the plotting of characteristic curves [54,55,56].

There are several models that are used for determining equilibrium distribution, but for the treatment of wastewater, the most commonly used models are the Freundlich and Langmuir isotherm models [54,56,57].

❖ Langmuir isotherm equation

The theory of Langmuir adsorption correlates the rapid lowering of attraction forces with the increase in distance between the molecules. This model has two parameters, as illustrated via Equation (4):

(4) $$q_{e}=\frac{q_{max}K_{L}C_{e}}{1+K_{L}C_{e}}$$

Equation (4) can be transformed into a linearized form, Equation (5):

(5) $$\frac{C_{e}}{q_{e}}=\frac{1}{K_{L}q_{max}}+\frac{C_{e}}{q_{max}}$$

where $q_{max}$ is the maximum adsorption capacity for forming a monolayer (mg g^−1^) and $K_{L}$ is the Langmuir constant (L mg^−1^).

From the Langmuir linear equation, $K_{L}$ and the $q_{max}$ were obtained from the intercept and the slope of the linearized plot of $C_{e}$*/*$q_{e}$ against $C_{e}$. For the description of the isotherm type, the dimensionless constant (the separation factor or the equilibrium parameter) ($R_{L}$) could be determined by applying Equation (6):

(6) $$R_{L}=\frac{1}{1+K_{L}C_{0}}$$

where $R_{L}$ shows the nature of sorption to be either unfavorable for $R_{L}$ > 1, linear for $R_{L}$ = 1, favorable for 0 < $R_{L}$< 1, or irreversible for $R_{L}$ = 0 [55,58].

❖ Freundlich isotherm equation

The Freundlich isotherm is the first formula that demonstrates reversible and non-ideal sorption, sorption which is not adequate for monolayer formation. It has two parameters, as indicated in Equation (7):

(7) $$q_{e}=K_{f}C_{e}^{\frac{1}{n}}$$

where $K_{f}$ is the Freundlich constant (mg g^−1^) (L mg^−1^) and n is the adsorption intensity.

Equation (7) can be transformed into a linearized form, Equation (8):

(8) $$\ln q_{e}=\ln K_{f}+\left(\frac{1}{n}\right)\ln C_{e}$$

The values of *1/n* and $K_{f}$ were obtained by plotting *ln* $q_{e}$ against *ln* $C_{e}$ as the slope and the intercept, respectively. Briefly, *1/n* (the intensity of the adsorption) predicts any phenomenon that happens along the process of adsorption; *1/n* < 1 means normal adsorption; *1/n* > 1 means cooperative adsorption; and *1/n* being near to zero means that the adsorbent surface is more heterogeneous [58].

### 4.8. Adsorption Kinetics

Exploring the adsorption kinetics is also useful for grasping the rate of adsorption on the adsorbent surface, because the adsorption kinetics exhibit the impact of the diverse conditions on the adsorption process’ quickness, applying models which describe the adsorption reaction. Moreover, the adsorption kinetics provide the mechanism of adsorption of the dyes by the adsorbents. 

The kinetic outcomes for the two investigated dyes (CR and CV) were modeled by employing two various kinetic models: pseudo-first-order, and pseudo-second-order models.

❖ Pseudo-First-Order Model (Lagergren Model)

This model is used to determine the relation between alterations in time and the capability of adsorption with an order of one. It is given via Equation (9), where k_1_ is the rate constant:

(9) $$\frac{\mathrm{d}q_{t}}{\mathrm{d}t}=k_{1}\left(q_{e}-q_{t}\right)$$

Integrating Equation (9) with the boundary conditions of *q_t_* = 0 at *t* = 0 and *q_t_* = *q_t_* at *t* = *t* gives a linear equation of the pseudo-first-order model, Equation (10):

(10) $$\log\left(q_{e}-q_{t}\right)=\log q_{e}-\frac{k_{1}}{2.303}t$$

The values of *q_e_* and *k*_1_ can be determined from the intercept and the slope of the linear plot of log (*q_e_* − *q_t_*) versus *t*.

❖ Pseudo-Second-Order Model (Ho and Mckay Model)

This model is given via Equation (11), which illustrates the relation between the capacity of adsorption and the concentration with a second order. It depicts the adsorption of the dissolved dye ions through chemical sharing or an exchange of cations on the surface of the adsorbents. This refers to a chemical reaction that occurs in the process of adsorption.

(11) $$\frac{\mathrm{d}q_{t}}{\mathrm{d}t}=k_{2}{\left(q_{e}-q_{t}\right)}^{2}$$

The integration of Equation (11) provides the linearized form of the pseudo-second-order equation: Equation (12).

(12) $$\frac{t}{q_{t}}=\frac{1}{k_{2}q_{e}^{2}}+\frac{t}{q_{e}}$$

where *k*_2_ is the pseudo-second-order constant (g mg^−1^ min^−1^). The slope and intercept of the linear plot of *t*/*q* against t yielded the values of *q_e_* and *k*_2_, respectively.

### 4.9. Kinetic Validation

For the determination of the most usable model to describe the process of adsorption, the normalized standard deviation (Δ*q_e_* (%) is determined and applied, employing Equation (13):

(13) $$\Delta q(\%)=100\;\times \;\sqrt{\frac{{\left[(q_{t,exp}-q_{t,cal})/q_{t,exp}\right]}^{2}}{N-1}}$$

where *q_t,exp_* is the experimental adsorption capacity (mg g^−1^), *q_t,cal_* is the calculated adsorption capacity for pseudo-first- and pseudo-second-order models (mg g^−1^), and *N* is the number of data points.

### 4.10. Desorption Study

The dye was desorbed from the adsorbent, firstly by rinsing it using distilled water to remove any non-adsorbed dye. Secondly, 10 mg of the adsorbent was individually soaked in 10 mL of various desorption media (EtOH, MeOH, MeCOMe, and a 0.1 N solution of NaOH) at 25 °C overnight. The quantity of the dye desorbed from the adsorbent could be determined by employing Equation (14):

(14) $$\%\;Dye\;desorption=q_{d}/q_{a}\times 100$$

where $q_{d}$ is the amount of dye desorbed from the adsorbent surface (mg g^−1^) and $q_{a}$ is the amount of dye adsorbed onto the adsorbent (mg g^−1^) [12].

## Figures and Tables

**Table 1:** The efficiency (%) of the prepared hydrogels and the H_31_/AgNP composites for CR dye, at 25 and 50 °C, at an initial dye concentration of 18 mg L ^−1^, and at pH 7.

Temperature	PVA	Chitosan	H_10_	H_11_	H_13_	H_31_	H_31_/AgNPs1%	H_31_/AgNPs3%	H_31_/AgNPs5%
25 °C	34.69	62.22	69.40	66.70	60.00	73.33	87.78	93.89	98.54
50 °C	37.50	84.03	96.80	90.30	76.57	99.61	99.61	99.62	99.91

**Table 2:** The removal efficiency (%) for CV dye of the prepared hydrogels and the H_31_/AgNP composites at 25 and 50 °C, at an initial dye concentration of 12 mg L^−1^, and at pH 7.

Temperature	PVA	Chitosan	H_10_	H_11_	H_13_	H_31_	H_31_/AgNPs1%	H_31_/AgNPs3%	H_31_/AgNPs5%
25 °C	13.75	5.42	11.17	15.17	17.50	9.28	42.05	46.62	58.04
50 °C	40.46	15.88	28.90	47.50	49.08	22.50	73.17	91.18	94.71

**Table 3:** Isotherm parameters obtained from Langmuir equation for the adsorption of the CV dye onto the prepared hydrogels and the H_31_/AgNP composites at 25 and 50 °C.

Temperature	Parameter	PVA	Chitosan	H_10_	H_11_	H_13_	H_31_	H_31_/AgNPs1%	H_31_/AgNPs3%	H_31_/AgNPs5%
25 °C	*q_max_* (mg g^−1^)	19.92	8.547	8.17	15.87	50.505	7.994	−9.804	−11.111	−21.277
	K_L_ (L mg^−1^)	0.009	0.007	0.02	0.013	0.0046	0.015	−26.422	−18.044	−21.979
	R^2^	0.216	0.324	0.474	0.578	0.132	0.22	0.088	0.462	0.524
	R_L_	0.981–0.898	0.985–0.919	0.962–0.808	0.974–0.861	0.991–0.948	0.971–0.849	−0.019–−0.003	−0.02– −0.005	−0.023–−0.004
50 °C	*q_max_* (mg g^−1^)	43.48	52.63	8.547	322.6	11.364	9.009	−1.828	−0.859	12.987
	K_L_ (L mg^−1^)	0.018	0.004	0.079	0.003	0.1833	0.046	−3.435	−1.408	0.247
	R^2^	0.178	0.010	0.872	0.215	0.891	0.853	0.558	0.067	0.757
	R_L_	0.966–0.826	0.993–0.957	0.863–0.513	0.994–0.967	0.732–0.313	0.915–0.643	−0.170–0.025	−0.551– −0.063	0.670–0.252

**Table 4:** Isotherm parameters obtained from Freundlich equation for the adsorption of the CV dye onto the prepared hydrogels and the H_31_/AgNP composites at 25 and 50 °C.

Temperature	Parameter	PVA	Chitosan	H_10_	H_11_	H_13_	H_31_	H_31_/AgNPs1%	H_31_/AgNPs3%	H_31_/AgNPs5%
25 °C	1/*n*	0.974	0.962	0.925	0.951	0.992	0.90	1.084	1.192	1.105
	*K_f_* (mg g^−1^)(L mg^−1^)	0.186	0.064	0.164	0.213	0.228	0.129	0.418	0.629	1.022
	R^2^	0.992	0.997	0.989	0.997	0.997	0.983	0.815	0.963	0.981
50 °C	1/*n*	0.95	0.992	0.78	0.993	0.69	0.86	2.505	2.787	0.529
	*K_f_* (mg g^−1^)(L mg^−1^)	0.752	0.195	0.68	0.924	0.5532	0.409	0.587	3.469	12.528
	R^2^	0.893	0.98	0.998	0.999	0.985	0.99	0.830	0.857	0.863

**Table 5:** The kinetic model constants and the correlation coefficients for the adsorption of the CR and CV dyes onto the hydrogels.

Kinetic Models	Parameters	CR	CV
		H_31_	H_13_
	*q_e.exp_* (mg g^−1^)	13.45	1.67
pseudo- first-order	R^2^	0.970	0.959
	*q_e.cal_* (mg g^−1^)	11.05	1.34
	K_1_ (10^−4^) (min^−1^)	1.74	1.74
	Δ*q_e_* (%)	5.15	5.70
pseudo- second-order	R^2^	0.980	0.990
	*q_e,cal_* (mg g^−1^)	12.00	1.67
	K_2_ (10^−4^) (g·mg^−1^·min^−1^)	4.28	45.77
	Δ*q_e_* (%)	3.11	0

**Table 6:** Performance characteristics of some adsorbents for the removal of CR dye.

Adsorbents	Adsorption Capacity, *q_max_* (mg/g)	pH	Initial Dye Concentration (mg/L)	Dosage of Adsorbent	References
Zeolite (clay materials)	3.77	7.5 ± 0.3	150.0	100 g/L	[38]
ZrO_2_ reagent	4.8 ± 0.2	7.0	35.0	400 mg/L	[33]
Natural kaolin (K)	5.94	6.9	100.0	0.1 g/10 mL	[39]
Australian kaolin clay K15GR	6.81	7.5 ± 0.3	200.0	50.0 g/L	[40]
Australian kaolin clay Ceram	7.27	7.5 ± 0.3	250.0	20.0 g/L	[40]
Nickel oxide nanoparticles	10.10	5.0	2.0	0.5 g/L	[41]
Montmorillonite	12.70	7.0	100	0.1 g/25 mL	[42]
H_13_	13.78	7	18	0.01 g/10 mL	This work
H_11_	16.247	7	18	0.01 g/10 mL	This work
H_31_	17.93	7	18	0.01 g/10 mL	This work
H_31_/AgNPs 5%	17.98	7	18	0.01 g/10 mL	This work
Polypyrrole	18.00	3.0	40.0	100 mg/50 mL	[43]
Raw rectorite (R-REC)	19.50	7.0	500.0	0.025 g/25 mL	[44]
ZrO2 solid spheres	21.4 ± 1.1	7.0	35.0	400 mg/L	[33]
Open burnt clay	22.86	3.0	50.0	0.5 g/200 mL	[45]
Cetyltrimethylammonium bromide-modified kaolin (CTAB-kaolin or KC)	24.46	6.9	100.0	0.1 g/10 mL	[39]
Raw clay	27.03	7.5 ± 0.2	50.0	1.0 g/100 mL	[46]

**Table 7:** Performance characteristics of some adsorbents for the removal of CV dye.

Adsorbent	*q_max_* (mg/g)	*C_o_* (mg/L)	pH	References
H_31_	2.7	12	7	This work
H_11_	5.7	12	7	This work
H_13_	5.89	12	7	This work
H_31_/AgNPs 5%	11.365	12	7	This work
Eichhornia crassipes live roots	20.84	1000	7	[47]
Jackfruit leaf powder (JLP)	43.39	100	7	[48]
NaOH-modified rice husk (NMRH)	44.87	50	8	[49]
Banyan leaf powder	81	150	8	[50]
Grapefruit peel	249.68	100	6	[51]
Treated ginger waste (TGW)	277.7	20	8	[52]
NaOH-activated Aerva javanica leaf (NAJL)	315.2	250	9	[53]

**Table 8:** Synthesis and elemental analyses of the prepared hydrogels.

Hydrogel Code	Chitosan: PVA (Weight Ratio)	Chitosan g (mmol)	PVA (g)	Trimellitic Anhydride Chloride g (mmol)	Ammonium Thiocyanate g (mmol)	Elemental Analysis (%)				
						C	H	N	O	S
Chitosan	-	-	-	-	-	45.10	6.77	8.43	39.70	-
PVA	-	-	-	-	-	54.54	9.10	-	36.36	-
H_10_	1:0	3 (18.63)	0	1.96 (9.32)	0.71 (9.32)	47.54	4.68	7.51	35.03	5.24
H_13_	1:3	1 (6.21)	3	0.65 (3.11)	0.24 (3.11)	52.79	8.00	1.88	36.02	1.31
H_11_	1:1	2 (12.42)	2	1.31 (6.21)	0.47 (6.21)	51.04	6.89	3.75	35.70	2.62
H_31_	3:1	3 (18.63)	1	1.96 (9.32)	0.71 (9.32)	49.29	5.79	5.63	35.36	3.93

## Data Availability

The data presented in this study are available on request from the corresponding authors.

## Supplementary Materials

The following supporting information can be downloaded at: https://www.mdpi.com/article/10.3390/gels9110882/s1.
