# Supplementary material for: Poly (Vinyl Alcohol) Hydrogels Boosted with Cross-Linked Chitosan and Silver Nanoparticles for Efficient Adsorption of Congo Red and Crystal Violet Dyes
Source: Gels. 2023 Nov 7;9(11):882. doi: 10.3390/gels9110882 (PMC10670830; doi:10.3390/gels9110882)
Supplement: Supplementary file 1 [file gels-09-00882-s001.zip › gels-2676389-supplementary.pdf]

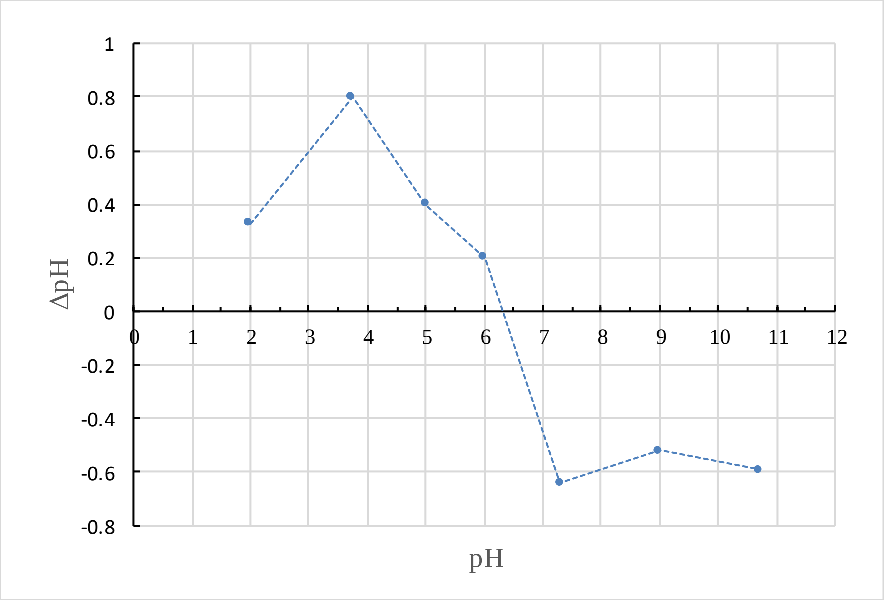

Online Supplemental File-S1
The pH of zero-point charge (pH_{zpc}) of H_{13}.

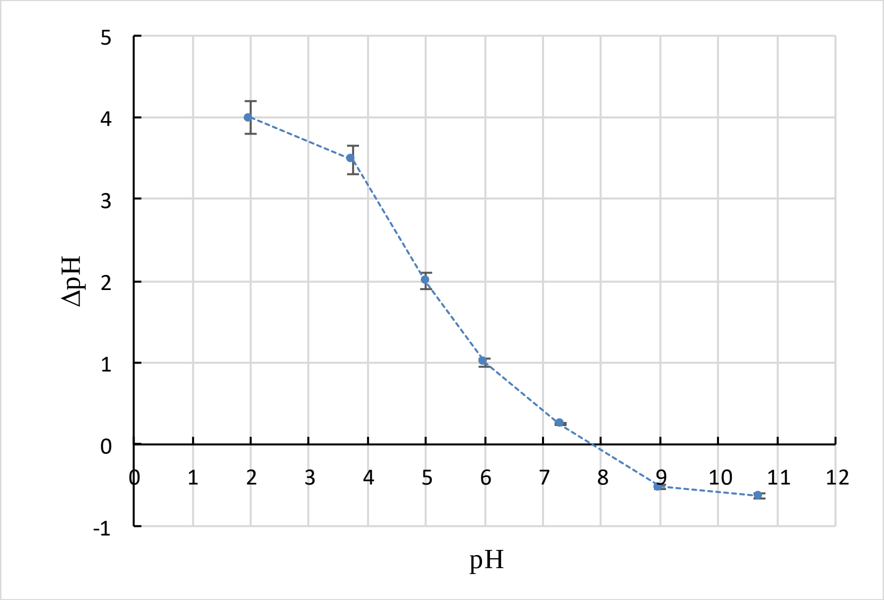

Online Supplemental File-S2
The pH of zero-point charge (pH_{zpc}) of H_{31}.

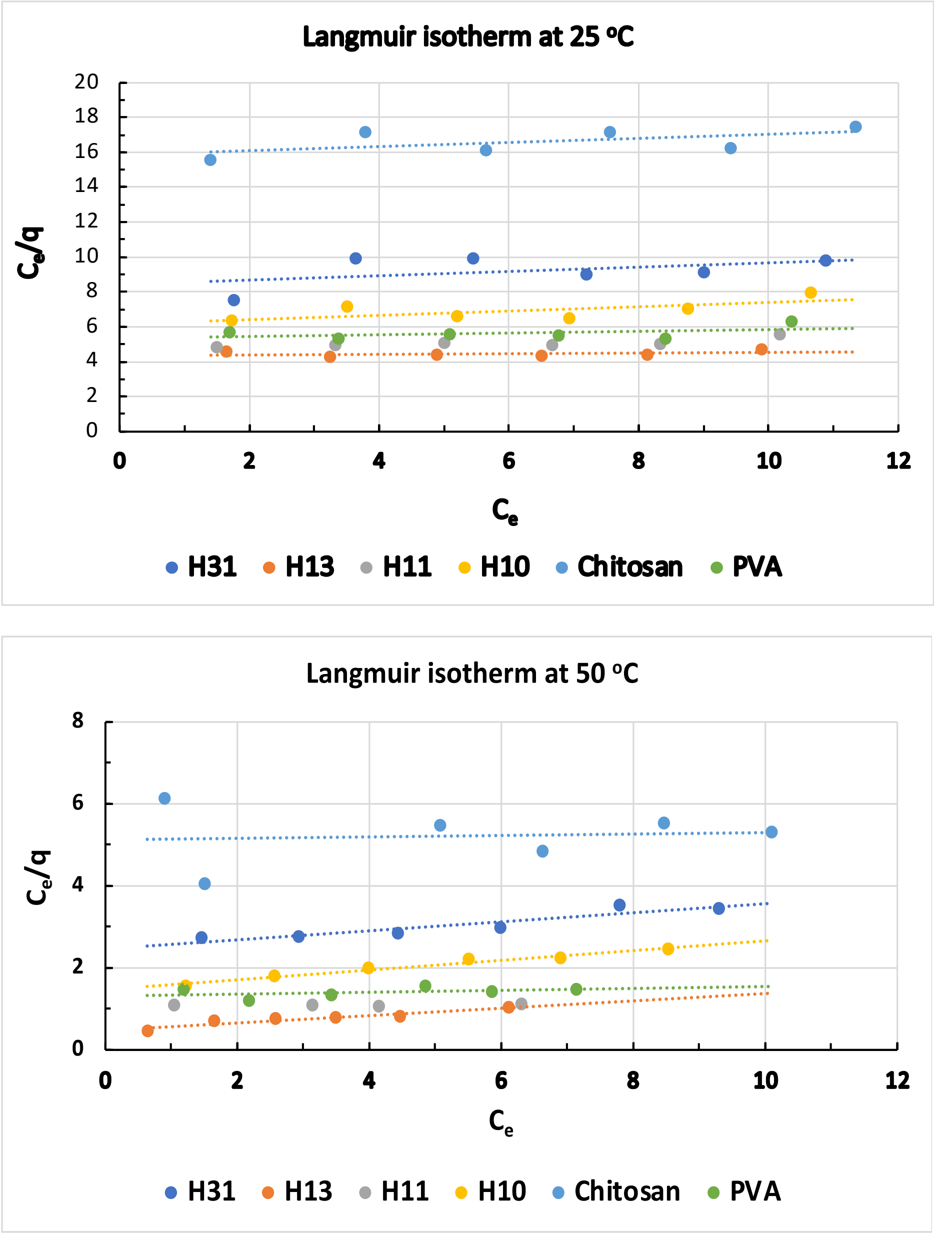

Online Supplemental File-S3
Langmuir isotherm of CV dye adsorption onto the prepared hydrogels.

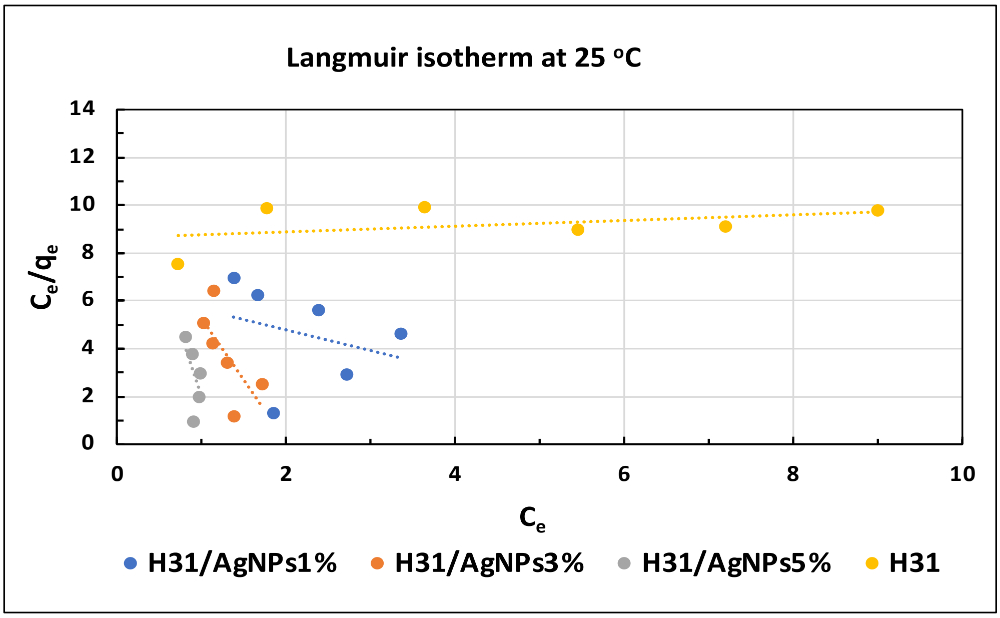

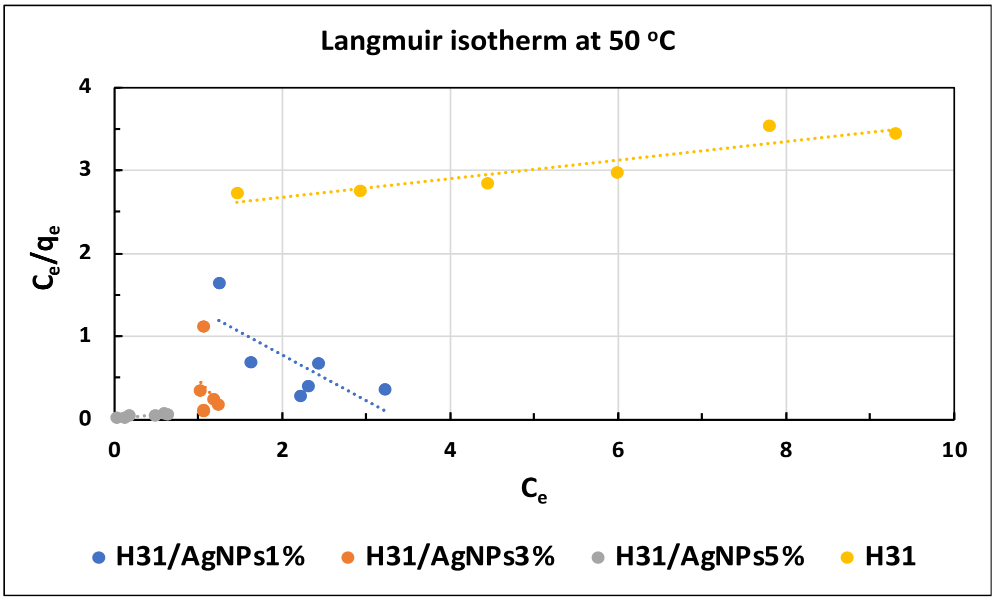

Online Supplemental File- S4

Langmuir isotherm of CV dye adsorption onto H_{31} and H_{31}/AgNP composites.

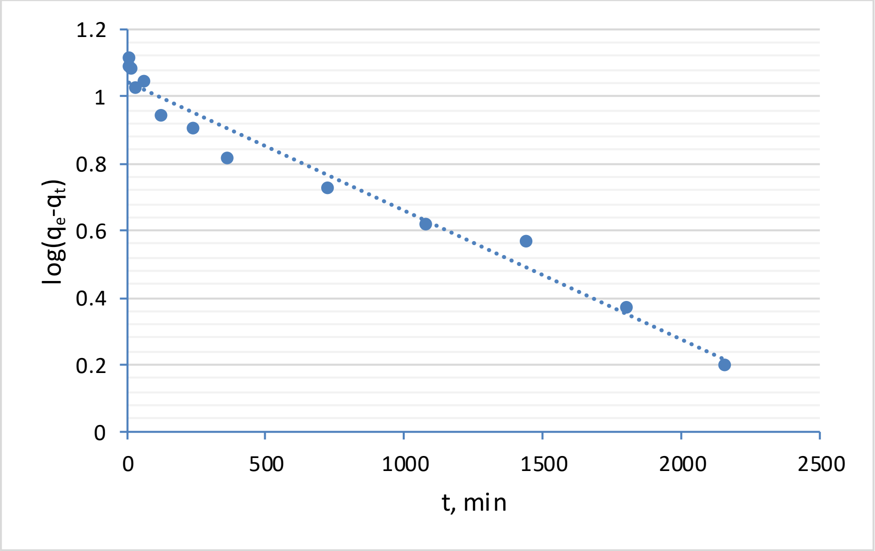

Online Supplemental File-S5
Linear regressions of the pseudo-first order kinetic model plot for the adsorption of CR dye onto H_{31}.

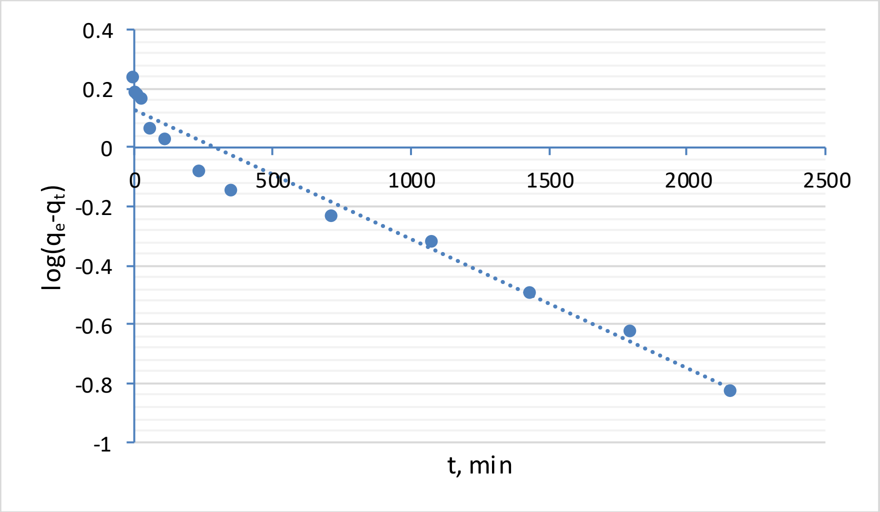

Online Supplemental File-S6
Linear regressions of the pseudo-first order kinetic model plot for the adsorption of CV dye onto H_{13}.

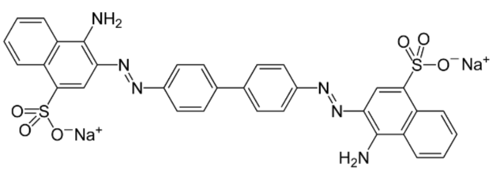

(a)

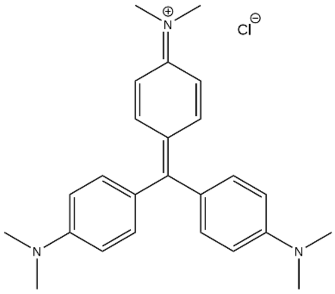

(b)

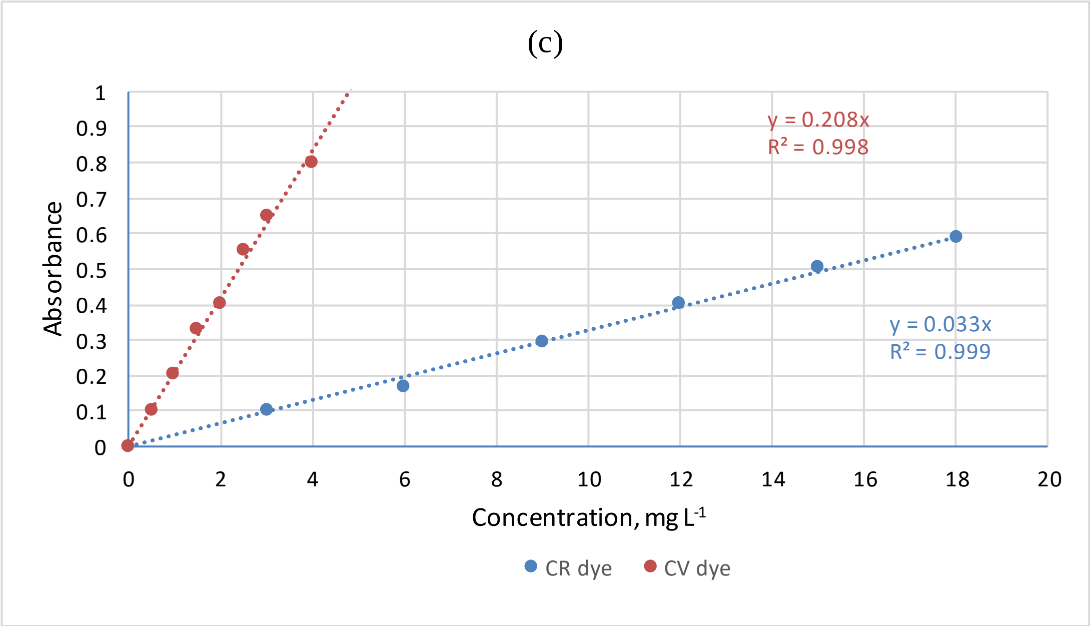

Online Supplemental File-S7
Chemical structure of (a) Congo Red (CR) and (b) Crystal Violet (CV) dyes, and (c) calibration curves.

1)

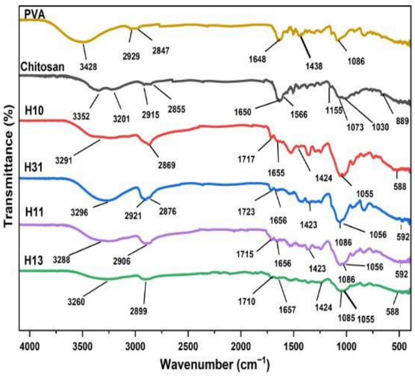

2)

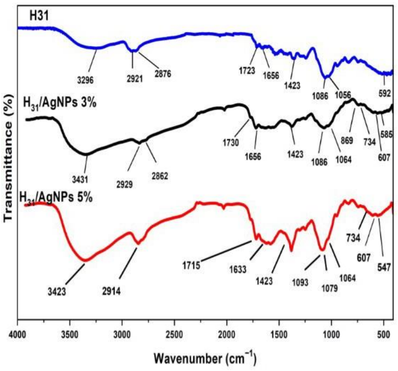

3)

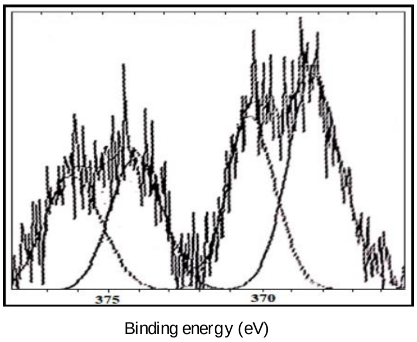

4)

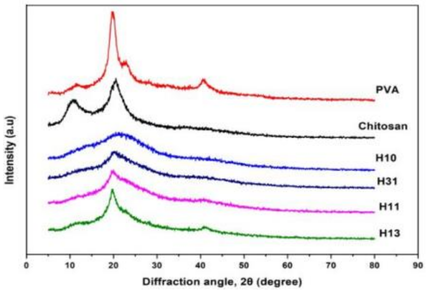

5)

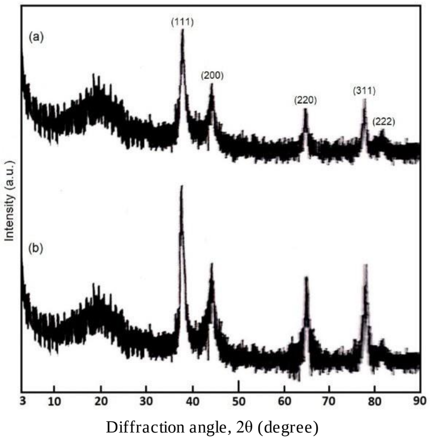

6)

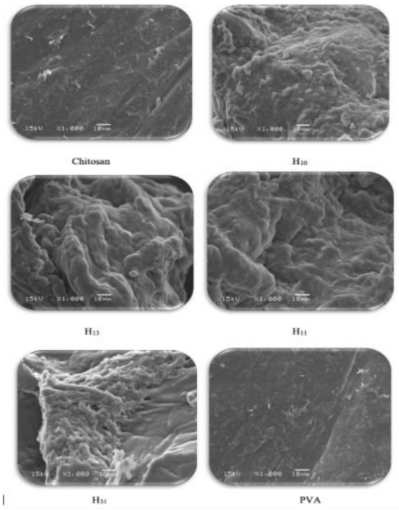

7)

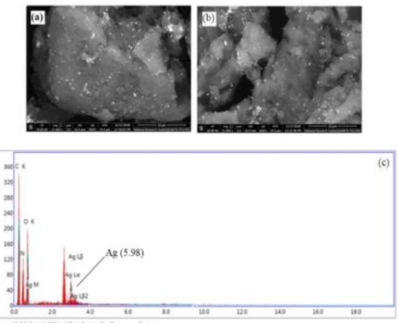

8)

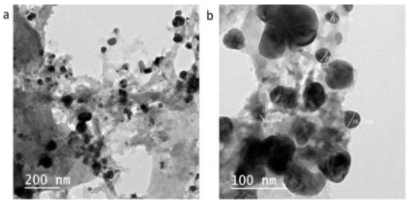

1) FTIR spectra of the prepared hydrogels.
2) FTIR spectra of H_{31} and H_{31}/AgNP composites.
3) XPS spectrum of H_{31}/AgNPs5% composite.
4) XRD patterns of the prepared hydrogels.
5) XRD pattern of: (a) H_{31}/AgNPs3% and (b) H_{31}/AgNPs5% composites.
6) SEM images of the prepared hydrogels.
7) SEM images of: (a) H_{31}/AgNPs1%; (b) H_{31}/AgNPs3% and (c) EDS of H_{31}/AgNPs3%.
8) TEM images of H_{31}/AgNPs5% composite at different magnifications: (a) 200 nm and (b) 100 nm.

Online Supplemental File-S8

Characterization of Chitosan/PVA Hydrogels and H_{31}/AgNP Composites.
